# The Pro-Survival Role of Autophagy Depends on Bcl-2 Under Nutrition Stress Conditions

**DOI:** 10.1371/journal.pone.0063232

**Published:** 2013-05-03

**Authors:** Hai-Dong Xu, Dan Wu, Jin-Hua Gu, Jian-Bin Ge, Jun-Chao Wu, Rong Han, Zhong-Qin Liang, Zheng-Hong Qin

**Affiliations:** Department of Pharmacology and Laboratory of Aging and Nervous Diseases, Soochow University School of Pharmaceutical Science, Suzhou, China; National University of Singapore, Singapore

## Abstract

Autophagy can be induced under nutrition stress conditions. Bcl-2 is a pro-survival protein which inhibits apoptosis and autophagy. However, the role of Bcl-2 in autophagy regulation and cell survival under nutrition deprivation has not been fully understood. This study sought to investigate if Bcl-2 upregulation is essential in limiting autophagic activity and prevent cell death under nutrition deprivation conditions. Autophagic activity was monitored by the changes in GFP-LC3 localization and protein levels of Beclin1, LC3-II, cathepsin D and p62 in neuroblastoma SH-SY5Y cells underwent serum deprivation. Manipulation of Bcl-2 function was achieved with siRNAs and small molecular inhibitors. The cell viability and apoptosis were assessed with MTT assay and Annexin V/PI staining. The results showed that serum starvation increased protein levels of LC3-II and Beclin1 but decreased autophagy substrate p62. Autophagy activation induced by serum deprivation and rapamycin was accompanied by an upregulation of Bcl-2 protein levels. When Bcl-2 was knocked down with siRNA or inhibited with HA 14-1 or ABT-737, serum starvation induced profound cell death and enhanced autophagic flux under nutrition deprivation conditions, while knockdown of autophagic gene Beclin1 or autophagy inhibitors (bafilomycin A1 and E64D), rescued cell death. In contrast, overexpression of Bcl-2 inhibited autophagy and blocked cell death in response to serum deprivation. These data suggest that Bcl-2 plays an essential role in limiting autophagy activation and preventing initiation of programmed cell death. Thus Bcl-2 may be an important mechanism for balancing beneficial and detrimental impacts of autophagy on cell survival.

## Introduction

Cellular homeostasis is dependent on the balance between biosynthesis and biodegradation. Macroautophagy, which is also referred to as autophagy, is an evolutionarily conserved pathway involving lysosome-dependent degradation of cytoplasmic materials [1], [2]. Autophagy begins with the sequestration and enclosure of part of cytoplasm by double-membrane vacuoles, called autophagosomes. Autophagosomes fuse with lysosomes where the luminal contents are degraded by lysosomal enzymes for recycling.

The role of autophagy in cell survival and cell death is controversial [3]. On the one hand, autophagy acts as an adaptive response to provide nutrients and energy on exposure to various stresses [4]. Removal of autophagy genes or pharmacologically blocking certain autophagic processes results in cell death [5]. In vivo study also suggests that autophagy genes are essential to maintain energy homeostasis during the early neonatal starvation period [6]. On the other hand, excessive or prolonged autophagy activation may promote cell death. Autophagy has long been proposed to be involved in type II programmed cell death, or autophagic cell death [7]. Early evidence showed that in conditions of defective apoptosis, such as bax^−/−^/bak^−/−^ murine embryonic fibroblasts (MEFs) treated with etoposide, or L929 cells treated with the caspase inhibitor Z-VAD, cell death were blocked by knockdown of essential autophagy genes [8], [9]. Other studies also point out that autophagy plays a role in cell death [10], [11]. Autophagy has been implicated in dead-cell clearance during programmed cell death (PCD) by the generation of energy-dependent engulfment signals [12]. Autophagy was also involved in the death of insulin-deprived neural stem cells [13], caspase-independent macrophage cells [14], and Drosophila larval salivary glands [15], [16]. Thus, the role of autophagy in cellular life and death is not a simple question.

The Bcl-2 family proteins are key regulators of apoptosis and autophagy. The founding member Bcl-2, which possesses four conserved Bcl-2 homology domains (BH1–4), suppresses apoptosis through its interaction with and sequestration of pro-apoptotic proteins, such as Bax and Bak [17]. Bax and Bak can oilgomerize into proteolipid pores and permeabilize the outer mitochondrial membrane, resulting in the release of cytochrome *c* and other intermembrane factors into the cytosol to initiate downstream apoptotic events [18], [19]. The ratio between the anti-apoptotic and pro-apoptotic Bcl-2 family members determine the sensitivity to apoptotic stimuli. Furthermore, anti-apoptotic Bcl-2 family proteins can bind the autophagy essential protein Beclin1 and inhibit Beclin1-dependent autophagy under acute starvation conditions [20]. The interaction between Bcl-2/Bcl-xl and Beclin1 is regulated by the proapoptotic BH3-only Bcl-2 family proteins [21] and the phosphorylation status of Bcl-2 protein mediated by c-Jun N-terminal kinase 1 [22]. Recently, Robert et al reported that knockdown of Bcl-B, a member of the Bcl-2 family of proteins, triggered cell death. They also found that the cell death was partially dependent on autophagy machinery [23]. However, autophagy induction has also been observed in Bcl-2 or Bcl-xl overexpressed models in response to ischemia [24] or apoptotic stimuli [8]. Thus, the precise role of anti-apoptotic protein Bcl-2 in starvation-induced autophagy activation and cell survival is not completely understood.

Our previous studies showed that autophagy was involved in neuronal cell death in excitotoxicity and ischemic brain damage [10], [11]. In these studies, we observed that autophagy activation was accompanied by a reduction in Bcl-2 protein levels. The decline in Bcl-2 protein levels was blocked by autophagy inhibitors. Suppression of Bcl-2 function with small molecular inhibitors further enhanced autophagic activity and cell death [25]. These studies suggest that there is a crosstalk between autophagy and apoptosis and Bcl-2 may play an important role in regulating both autophagy and apoptosis. In this study, we utilized a classical autophagy activation model with serum starvation to evaluate the role of Bcl-2 in modulating autophagy flux and cell survival under nutrition stress conditions. Our data indicate that Bcl-2 plays an essential role in limiting autophagy over-activation and preventing autophagic and apoptotic cell death under nutrition deprivation conditions.

## Results

### Serum Deprivation-induced Autophagy was Associated with a Bcl-2 Upregulation

**Figure 1:**
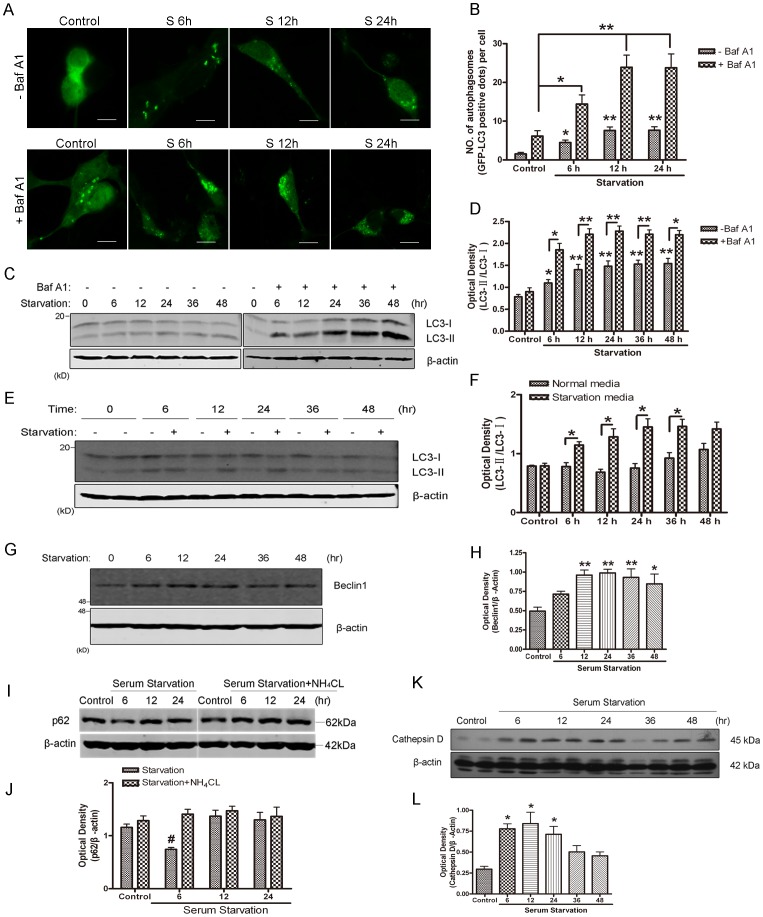
Autophagy was induced in SH-SY5Y neuroblastoma cells by serum deprivation. A: Representative confocal images (5 µM scale bar) of GFP-LC3 in SH-SY5Y cells transfected with GFP-LC3 plasmid. Twenty-four hours after transfection, cells were treated with serum-free medium in the presence or absence of 100 nM Baf A1 for the indicated times. B: Quantification of autophagy in serum-starved SH-SY5Y cells transfected with GFP-LC3. Data represent mean ± SD for combined data from three independent experiments. C: Western blot analysis of LC3 expression in SH-SY5Y cells subjected to serum-free medium in the presence or absence of 100 nM Baf A1. E: Western blot analysis of LC3 expression in SH-SY5Y cells subjected to serum-free medium. Each time course has a control. D and F: Quantitative analysis of optical densities of LC3-II/LC3-I with Sigma Scan Pro 5. Bars represent Mean ± SD (n = 3). G and H: Western blot analysis (G) and quantitative analysis (H) of Beclin1 expression in SH-SY5Y cells subjected to serum-free medium. Bars represent Mean ± SD (n = 5). I and J: Western blot analysis (I) and quantitative analysis (J) of p62 expression in SH-SY5Y cells subjected to serum-free medium with and without NH_4_Cl. Bars represent Mean ± SD (n = 3). K and L: Western blot analysis (K) and quantitative analysis (L) of cathepsin D expression in SH-SY5Y cells subjected to starvation. Bars represent Mean ± SD (n = 5). The optical densities of each protein band were quantified with Sigma Scan Pro 5 and normalized to the loading control. Statistical analysis was carried out with ANOVA followed by Dunnett t-test. (B and D) *p<0.05 and **p<0.01 vs control group. (I) #p<0.05 represent starvation vs starvation+NH_4_Cl.

Autophagy can be induced by starvation. To confirm if the serum-free medium induced autophagy, the human SH-SY5Y neuroblastoma cells were treated with serum-free medium for different time courses and the changes in the levels of autophagy regulatory proteins were determined. Serum starvation caused overall reduction in cell size but no robust cell death as observed with inverted microscopy (data not shown). The microtubule-associated protein 1 light chain 3 (LC3) was initially identified as a protein co-purified with microtubule-associated protein A1 and B1 from rat brain [26]. LC3 is a homologue of Apg8p essential for autophagy in yeast [27]. The LC3-II is localized in autophagosomes to help elongation of autophagosomal membranes [28]. Thus, LC3-II has been defined as a marker of autophagosomes in mammalian cells. To monitor the formation of autophagosomes, the SH-SY5Y cells were transfected with GFP-LC3 for 24 hours, the cells were then treated with serum-free medium for various length of time as indicated. As shown in figure 1, serum deprivation induced the redistribution of GFP-LC3 from a diffuse pattern to punctate structures (Fig. 1, A and B). To detect autophagic flux, bafilomycin A1 (Baf A1, a potent and specific inhibitor of vacuolar H^+^ ATPase [29]), was used to inhibit the acidification of the lysosome and autophagosome-lysosome. Baf A1 significantly enhanced GFP-LC3 patches under serum deprivation conditions (Fig. 1A). Western blot analysis of LC3 also observed that the upregulation of LC3-II induced by serum deprivation dramatically accumulated in the presence of Baf A1 (Fig. 1, C and D). These results indicate that autophagy is stimulated by serum starvation. Autophagy is sensitive to and can be influenced by many other factors. In order to exclude the possibility that the changes in LC3-II levels were caused simply by medium change, we harvested samples with a control at each time point. The data showed that there was a decrease in LC3-I and an increase in LC3-II at each time point examined, as compared with their respective controls (Fig. 1, E and F), indicating that autophagy was indeed induced. After serum deprivation, Beclin1 was also up-regulated (Fig. 1, G and H). Beclin1, the mammalian orthologue of Atg6, promotes autophagy in human breast carcinoma MCF-7 cells [30], by localizing at the trans-Golgi network and participates in the initial steps of vesicle nucleation through forming a complex with the class III phosphatidylinositol 3-kinase (PI3K) [31]. p62/sequestosome 1 (SQSTM1) is a protein involved in the formation of autophagosomes which is constitutively degraded by the autophagic pathway through specific binding to LC3 [32], [33]. Serum starvation also resulted in a transient down-regulation of p62 at 6 hours, followed by a quick recovery at other time points (Fig. 1I). To further assess autophagic flux, we treated the cells with NH_4_CL, an inhibitor of lysosomal acidification that can inhibit autophagic degradation of autophagic substrates. Addition of NH_4_CL recovered the levels of p62 at 6 hours, indicating p62 was indeed degraded by the autophagy process (Fig. 1, I and J). Autophagy is a constitutive process involving activation of lysosomal enzymes and subsequent degradation of substrates [34]. Therefore, we measured levels of cathepsin D, an aspartic protease localized inside the lysosomes. Cathepsin D was up-regulated upon serum starvation (Fig. 1, K and L). Overall, these data demonstrated that serum starvation enhanced autophagy flux, led to increase in autophagosome formation, activation of lysosomes and degradation of autophagic substrates.

**Figure 2:**
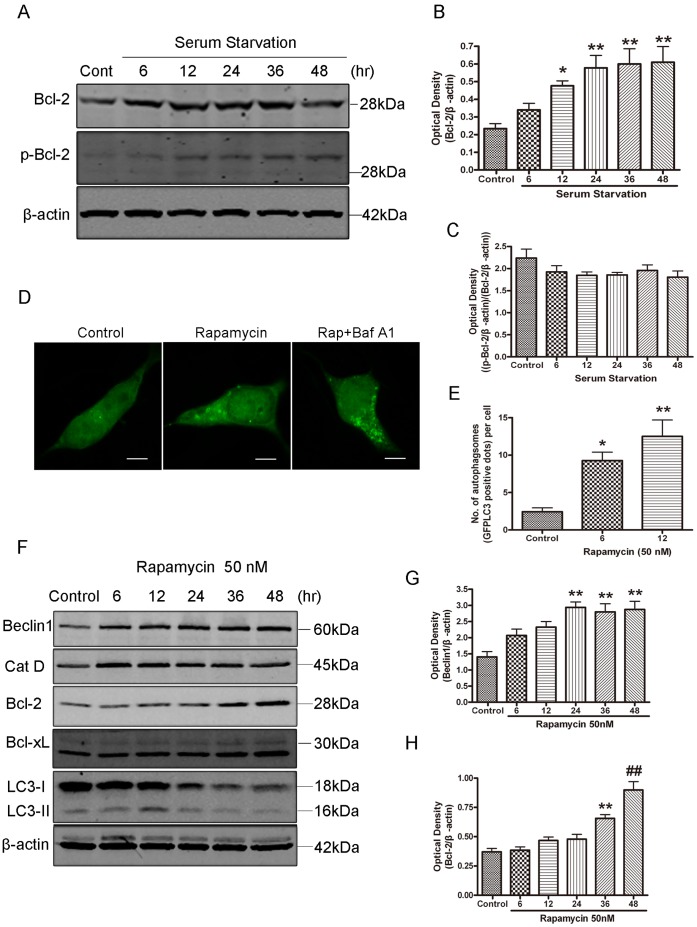
Bcl-2 was up-regulated in SH-SY5Y cells during serum starvation. A: Western blot analysis of Bcl-2 and p-Bcl-2 expression in SH-SY5Y cells subjected to starvation. B and C: Quantitative analysis of optical densities of the Bcl-2 and p-Bcl-2 protein bands with Sigma Scan Pro 5 and normalized to the loading control. Bars represent Mean ± SD (B, n = 7; C, n = 3). D: Representative confocal images (5 µm scale bar) of GFP-LC3 assay in SH-SY5Y cells transfected with GFP-LC3 plasmid. Twenty four hours after transfection, the cells were treated with rapamycin (50 nM) for 12 hrs in the present or absence of 100 nM Baf A1. E: Quantification of autophagy in rapamycin-treated SH-SY5Y cells transfected with GFP-LC3. Data represent mean ± SD from three independent experiments. F: Western blot analysis of Beclin1, Cathepsin D, Bcl-2, Bcl-xl and LC3 in SH-SY5Y cells treated with Rapamycin. G and H: Rapamycin induced upregulation of Beclin1 and Bcl-2 in SH-SY5Y cells. Quantitative analysis of optical densities of the Beclin1 and Bcl-2 protein bands with Sigma Scan Pro 5 and normalized to the loading control. Bars represent Mean ± SD (G, n = 3; H, n = 4). Statistical analysis was carried out with ANOVA followed by Dunnett t-test. *p<0.05 vs control group; **p<0.01 vs control group; ##p<0.001 vs control group.

Previous studies have shown that Bcl-2 can bind essential autophagic protein Beclin1 and inhibit autophagy activation under acute nutrient deprivation conditions [20]. The present study found that serum starvation induced the up-regulation of total and phosphorylated Bcl-2 (Fig. 2, A and B). However, quantitative analysis of the ratio between phosphorylated Bcl-2 and total Blc-2 had no significant change (Fig. 2C). Previous studies have shown that phosphorylated Bcl-2 localizes predominantly to the ER [35], and that starvation can induce phosphorylation of the ER-localized pool of Bcl-2 [22]. The present study is consistent with the previous observation that Bcl-2 was phosphorylated under acute starvation conditions [22]. This study found that total Bcl-2 protein levels were up-regulated, thus, the increase of phosphorylated Bcl-2 could be due to the increased pool of total Bcl-2. To further test if an increase in autophagy activity through inhibition of mammalian target of rapamycin (now called the mechanistic target of rapamycin within higher eukaryotes) would cause an up-regulation of Bcl-2, the present study also used rapamycin to induce autophagy in SH-SY5Y cells. Rapamycin is a putative autophagy activator which inhibits mTOR and mimics nutrient deprivation condition in yeast, Drosophila, and mammalian cells [36], [37]. The present results showed that rapamycin treatment induced an increase in punctate structures in SH-SY5Y cells transfected with GFP-LC3 plasmid (Fig. 2, D and E). Moreover, more number of GFP-LC3 patches accumulated in the presence of Baf A1 (Fig. 2D). Other autophagy regulatory proteins were also detected after rapamycin treatment. LC3 was slightly increased at 12 hours, following a decrease at 24 and 48 hours. This may be due to the continuous activation and degradation of autophagosomes, which depleted the LC3 protein pool in the cytosol. Cathepsin D was persistently increased (Fig. 2F). Rapamycin also induced a significant upregulation of Beclin1 (Fig 2, F and G) and Bcl-2 (Fig. 2, F and H), suggesting autophagy activation is accompanied by a Bcl-2 up-regulation in these conditions.

### Serum Deprivation Induced Cell Death when Bcl-2 Function was Suppressed

**Figure 3:**
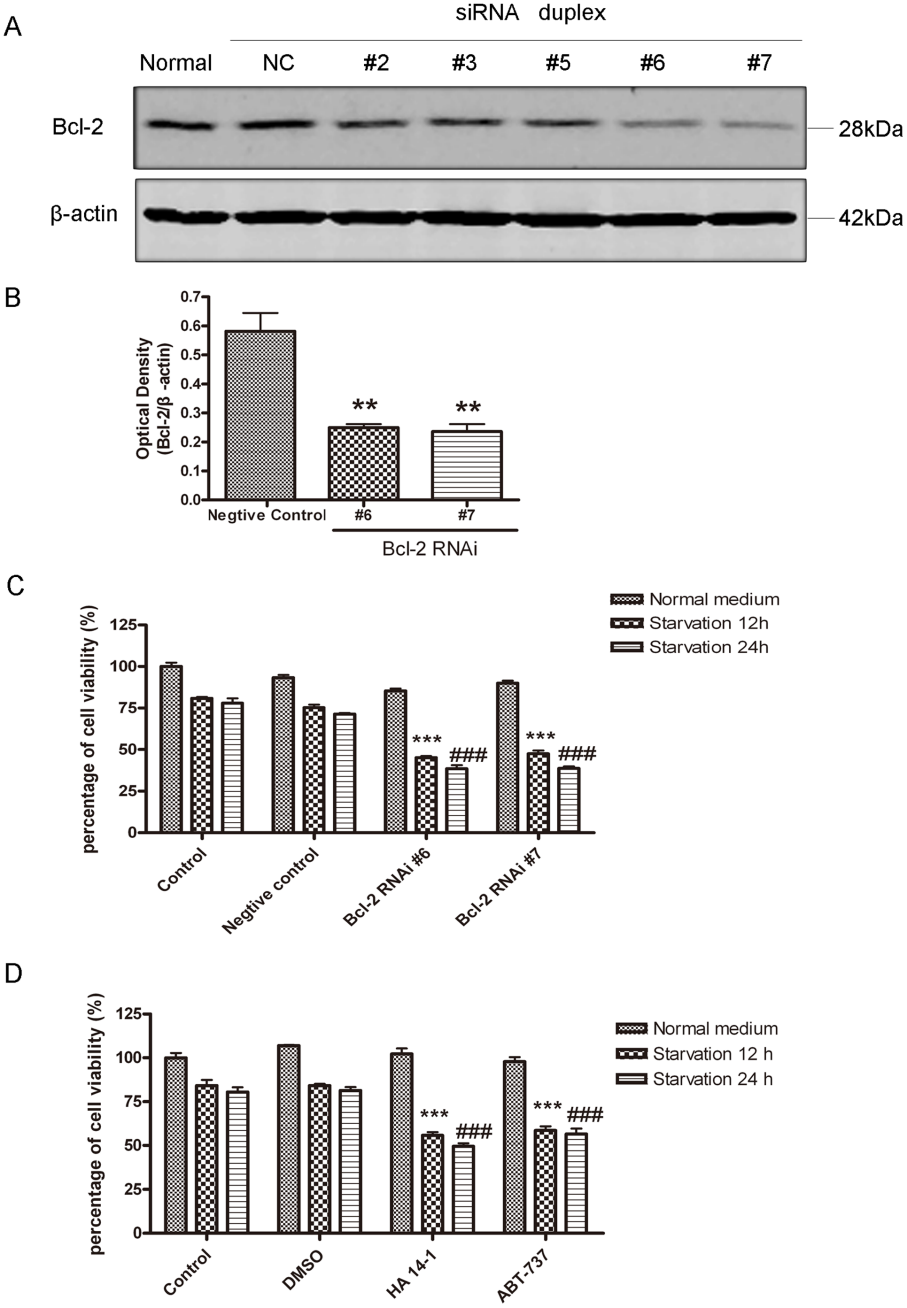
Nutrient deprivation decreased cell viability when Bcl-2 was down-regulated. A: Western blot analysis of Bcl-2 expression in SH-SY5Y cells after transfection with negative control and Bcl-2 siRNA oligonucleotides. B: Quantitative analysis of optical densities of Bcl-2 protein bands (NC, negative control; Bcl-2 RNAi #6 and Bcl-2 RNAi #7) and normalized to the loading control. Bars represent Mean ± SD (n = 4). Statistical analysis was carried out with ANOVA followed by Dunnett t-test. **p<0.01 represent Bcl-2 RNAi groups (#6 and #7) vs negative control group. C: Quantitative analysis of cell viability with MTT assay in SH-SY5Y control cells and the cells after transfection with NC, #6 and 7# oligonucleotides. Cells were either maintained in normal medium or subjected to 12 hrs or 24 hrs of starvation prior to cell viability assay. ***p<0.001 represent the indicated groups vs NC starvation 12 hrs group; ###p<0.001 represent the indicated groups vs NC starvation 24 hrs group. D: Quantitative analysis of cell viability with MTT assay in SH-SY5Y control cells and the cells treated with DMSO, HA 14-1 and ABT-737. After pre-treated with HA 14-1 or ABT-737, cells were maintained either in normal medium or starvation medium containing DMSO, HA14-1 and ABT-737 for 12 hrs or 24 hrs prior to cell viability assay. Statistical analysis was either carried out with Student t-test, or carried out with ANOVA followed by Dunnett t-test. ***p<0.001 represent HA 14-1/ABT-737 starvation 12 hrs groups vs DMSO starvation 12 hrs group; ###p<0.001 represent HA 14-1/ABT-737 starvation 24 hrs groups vs DMSO starvation 24 hrs group. For C and D, data represent mean ± SD for combined data from three independent experiments, each experiment has six replicate wells.

Bcl-2 is a well-defined anti-apoptotic protein, which also inhibits autophagy. Therefore, we reasoned that the role of upregulated Bcl-2 is either to protect the cells from death, or prevent the overactivation of autophagy. To evaluate the actual role of Bcl-2 in serum deprivation-induced autophagy, Bcl-2 was knocked down in SH-SY5Y cells with siRNA. Among many siRNA duplexes tested, the number six and number seven acquired a ∼60% silencing efficiency (Fig. 3, A and B). We investigated whether Bcl-2 knockdown affected the survival of SH-SY5Y cells under starvation conditions. While serum deprivation (12 hrs and 24 hrs) slightly decreased cell viability in all treated groups, the cell viability was greatly reduced in nutrient-starved Bcl-2 siRNA groups (e.g., #6 and #7) as compared with nutrient-starved negative siRNA-treated control group (Fig. 3C). We also used two small molecular antagonists of Bcl-2, HA 14-1 and ABT-737, to inhibit the function of Bcl-2. The treatment of these two compounds did not significantly change cell viability in control and DMSO groups during growth in normal conditions. In contrast, nutrient deprivation (12 hrs and 24 hrs) decreased cell viability in HA 14-1 or ABT-737 pre-treatment groups (Fig. 3D). These data suggest that Bcl-2 plays an essential role to maintain the viability of SH-SY5Y cells under serum deprivation conditions.

**Figure 4:**
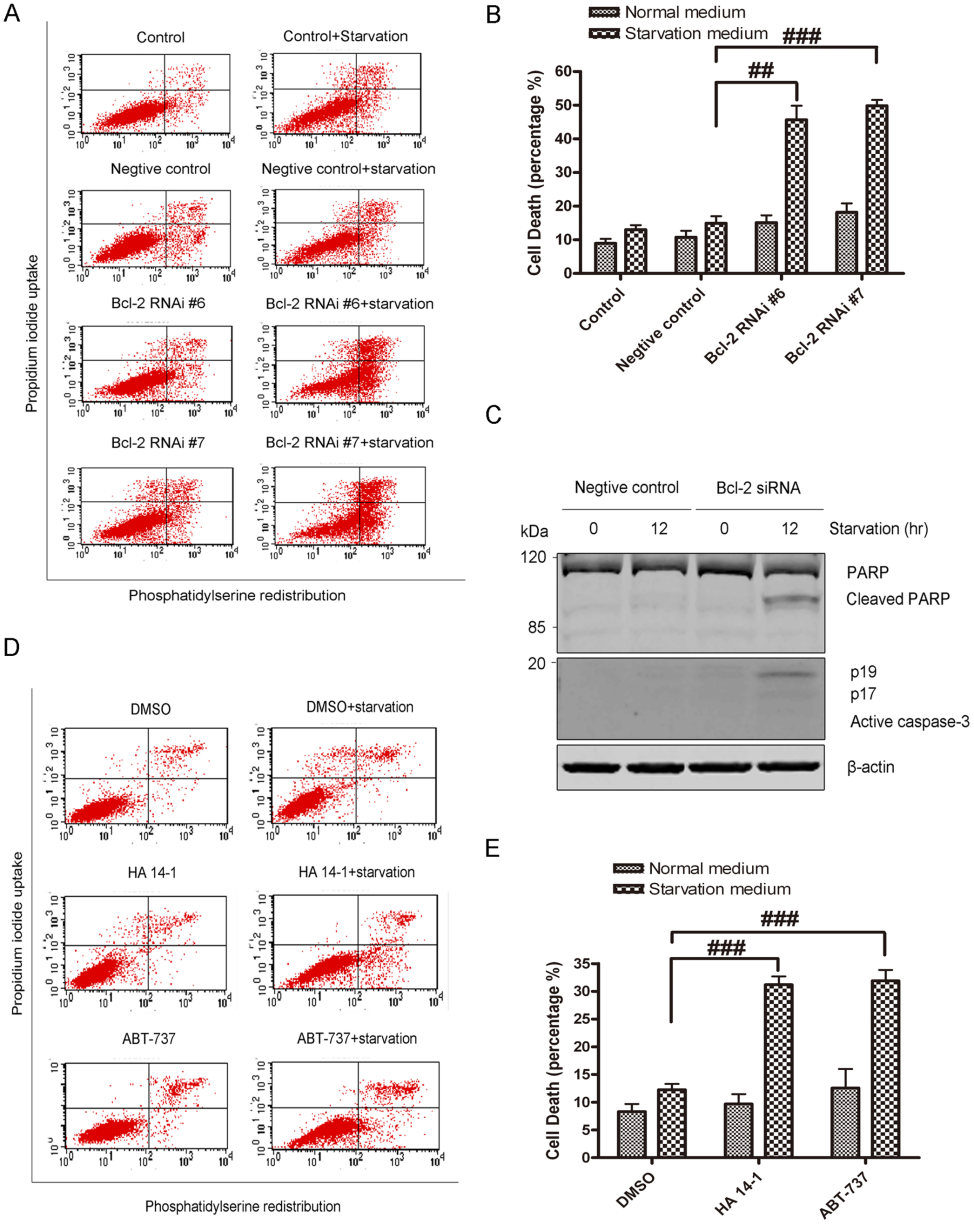
Serum deprivation induced cell death when Bcl-2 function was suppressed. A: Flow cytometry analysis of SH-SY5Y control cells transfected with negative control, Bcl-2 RNAi #6 and Bcl-2 RNAi #7. The cells were either growing in normal medium or subjected to 12 hrs starvation. B: Quantitative analysis of cell death by flow cytometry Annexin V/PI staining. Cells were either maintained in normal medium or subjected to 12 hrs of starvation prior to cell death analysis. Statistical analysis was either carried with Student t-test or carried out with ANOVA followed by Dunnett t-test (##p<0.01, ###p<0.001, Bcl-2 RNAi #6 + starvation, or #7 + starvation group vs NC+starvation group). C: Nutrient deprivation induced activation of caspase-3 (p19 and p17) and PARP cleavage in SH-SY5Y cells when Bcl-2 was knocked down. The results are representative of three experiments. D: Flow cytometry analysis of SH-SY5Y.control cells treated with DMSO, HA 14-1 and ABT-737. E: Quantitative analysis of cell death by flow cytometry Annexin V/PI staining in SH-SY5Y cells treated with DMSO, HA 14-1 and ABT-737. Cells were either maintained in normal medium or subjected to 12 hrs of starvation prior to cell death analysis. Statistical analysis was either carried out with Student t-test or carried out with ANOVA followed by Dunnett t-test (###p<0.001, HA 14-1 + starvation, or ABT-737 + starvation group vs DMSO + starvation group). For B and E, data represent mean ± SD for combined data from three independent experiments.

To test if nutrient deprivation induces apoptotic cell death when the function of Bcl-2 is inhibited, the cells were stained with Annexin V and PI and subjected to flow cytometry examination. Culture cells in starvation medium induced minor cell death. In contrast, cell death of the Bcl-2 siRNA groups significantly increased comparing with negative siRNA-treated control group (Fig 4, A and B). Furthermore, the active forms of caspase-3 and cleaved PARP (Fig. 4C) were detected in nutrient-starved SH-SY5Y cells when Bcl-2 was knocked down, indicating that apoptotic cell death was induced. The present study also examined apoptotic cell death of SH-SY5Y cells after treatment with HA 14-1, ABT-737 and DMSO (vehicle control). HA 14-1 and ABT-737 did not induced substantial cell death, but when the function of Bcl-2 was inhibited by the inhibitors, nutrient deprivation led to significantly more cell death as compared with the DMSO treatment group (Fig. 4D and E). These data suggest that Bcl-2 partially blocks the apoptotic cell death of SH-SY5Y cells under nutrient deprivation conditions.

### Autophagic Flux was Enhanced when Bcl-2 was Inhibited

**Figure 5:**
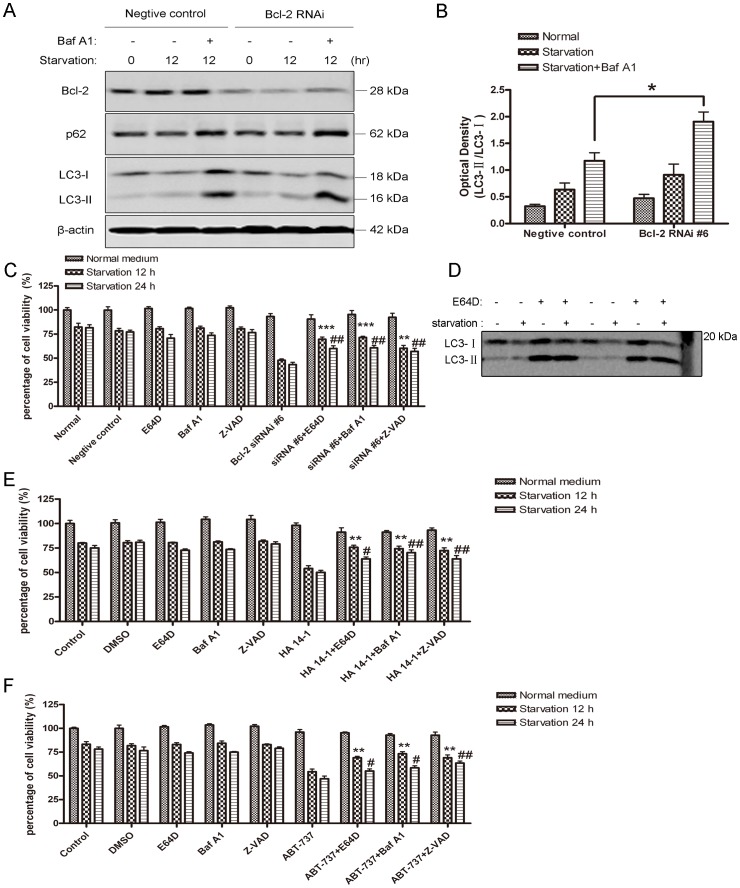
Autophagic flux induced by serum deprivation was enhanced when Bcl-2 was down-regulated and inhibitors of autophagy and apoptosis rescue cell from death. A: Western blot analysis of LC3-II and p62 expression in SH-SY5Y cells. Seventy-two hours after transfection with indicated oligonucleotides, the cells were subjected to starvation for 12 hrs in the present or absence of 50 nM Baf A1. B: Quantitative analysis of optical densities of the LC3-II/LC3-I protein bands with Sigma Scan Pro 5. Bars represent Mean ± SD (n = 3). Statistical analysis was carried out with unpaired t-test (*p<0.05, Bcl-2 siRNA #6 group vs negative control group). C: Quantitative analysis of cell viability with MTT assay. SH-SY5Y cells were treated with Bcl-2 siRNA #6 for 72 hrs, and then were treated with 10 µg/ml E64D, 50 nM Baf A1 and 50 µM Z-VAD. Cells were either maintained in normal medium or subjected to 12 hrs or 24 hrs of starvation prior to cell viability assay. **p<0.01, ***P<0.001 represent the indicated groups vs Bcl-2 RNAi #6 starvation 12 hrs group; ##p<0.01 represent the indicated groups vs Bcl-2 RNAi #6 starvation 24 hrs group. D: Representative western blot image of LC3 in SH-SY5Y cells subjected to normal or serum-free medium in the presence or absence of 10 µg/ml E64D for 12 hrs. E and F: Quantitative analysis of cell viability with MTT assay. SH-SY5Y cells were pre-treated with HA 14-1 or ABT-737, then the cells were either maintained in normal or starvation medium for 12 hrs or 24 hrs with E64D, Baf A1 or Z-VAD. **p<0.01 represent the indicated groups vs HA 14-1/ABT-737 starvation 12 hrs groups; #p<0.05, ##p<0.01 represent the indicated groups vs HA 14-1/ABT-737 starvation 24 hrs groups. Statistical analysis was either carried out with Student t-test, or carried out with ANOVA followed by Dunnett t-test. Data represent mean ± SD for combined data from three independent experiments, each experiment has six replicate wells.

To investigate the dynamic process of autophagic flux induced by serum deprivation when Bcl-2 is down-regulated, SH-SY5Y cells were treated with serum-free medium after Bcl-2 siRNA treatment, and then autophagosome marker LC3-II was determined in the presence of Baf A1. As the data shown, a conversion of LC3-I to LC3-II induced by serum starvation was robustly accumulated in the presence of Baf A1 in both negative control and Bcl-2 siRNA groups (Fig. 5A). However, when Bcl-2 was knocked down, the ratio of LC3-II/LC3-I was further enhanced compared with the negative siRNA-treated control group (Fig. 5B), indicating that starvation-induced autophagic flux was further increased when Bcl-2 was inhibited. In addition, autophagy substrate p62 was further accumulated in the presence of Baf A1 compared with the control group, consistent with the enhanced autophagic flux after down-regulation of Bcl-2. These data indicate that serum starvation induced autophagy can be further enhanced when Bcl-2 is knocked down.

### Inhibitors of Autophagy and Apoptosis Reduced Starvation-induced Cell Death Result from Bcl-2 Downregulation

Although serum deprivation induced apoptotic cell death when Bcl-2 was knocked down, autophagy was also further increased in this process. To evaluate whether over-activity of autophagy plays a role in the process of cell death, we used several inhibitors of autophagy-lysosome pathway to block autophagy, and measured the changes in cell viability. Consistent with the previous results, nutrient starvation (12 hrs and 24 hrs) slightly decreased cell viability in normal control and DMSO-treated control groups. When Bcl-2 was knocked down, the cell viability greatly decreased. Baf A1 and E64D (inhibitors of lysosomal proteases) alone did not change cell viability under normal or starvation conditions. However, these two inhibitors rescued cell death in the conditions that either Bcl-2 was knocked down by siRNA duplexes or its function was inhibited by HA 14-1 or ABT-737 (Fig. 5, C, E and F). To detect whether E64D at this concentration indeed blocked autophagy pathway, the SH-SY5Y cells were maintained in normal or starvation medium in the presence of absence of 10 µg/ml E64D (Fig. 5D). Both LC3-I and LC3-II significantly accumulated in the presence of E64D under normal or starvation conditions. The accumulated LC3-II was caused by inhibition of autophagic degradation process by E64D. We also used Z-VAD, a pan-caspase inhibitor, to block apoptosis and found that cell death was partially rescued, indicating that cells also died from a caspase-dependent apoptotic pathway. These data suggest that both autophagic and apoptotic mechanisms contribute to serum deprivation-induced cell death when Bcl-2 is down-regulated.

### Knockdown of Beclin1 Rescued Bcl-2 Knockdown-induced Cell Death in Response to Starvation, while Overexpression of Bcl-2 Blocked Cell Death

**Figure 6:**
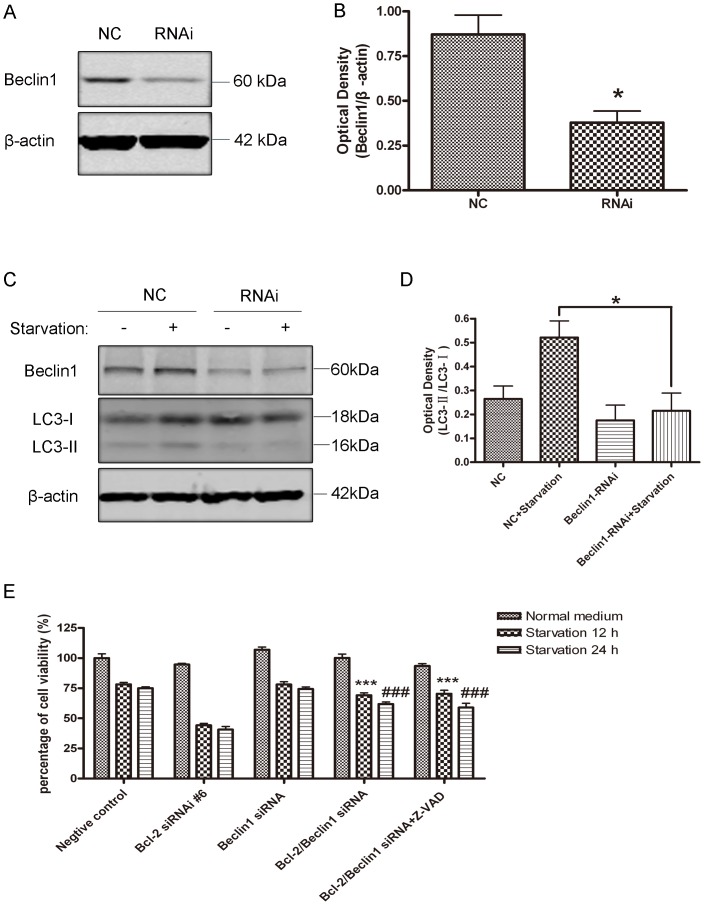
Knockdown of Beclin1 rescued Bcl-2 knockdown-induced cell death in response to starvation. A and B: Western blot analysis of Beclin1 expression in SH-SY5Y cells 72 hr after transfection and quantitative analysis of optical densities of Beclin1 protein bands with Sigma Scan Pro 5 and normalized to the loading control (B). Bars represent Mean ± SD (n = 3). Statistical analysis was carried out with ANOVA followed by Dunnett t-test (*p<0.05). C and D: After Beclin1 was knocked down, the cells were subjected to serum starvation for another 12 hrs, and then Beclin1 and LC3 were detected (C) and quantitative analysis of optical densities of the LC3-II/LC3-I protein bands with Sigma Scan Pro 5 (D, **p<0.01). Bars represent Mean ± SD (n = 3). C: Quantitative analysis of cell viability with MTT assay. SH-SY5Y cells were co-transfected with Bcl-2, Beclin1 or Bcl-2/Beclin1 siRNA for 72 hrs, and the cells were subjected to serum starvation for 12 hrs or 24 hrs in the presence or absence of 50 µM Z-VAD. ***P<0.001 represent the indicated groups vs Bcl-2 RNAi #6 starvation 12 hrs group; ###p<0.001 represent the indicated groups vs Bcl-2 RNAi #6 starvation 24 hrs group. Statistical analysis was either carried out with Student t-test, or carried out with ANOVA followed by Dunnett t-test. Data represent mean ± SD for combined data from three independent experiments, each experiment has six replicate wells.

**Figure 7:**
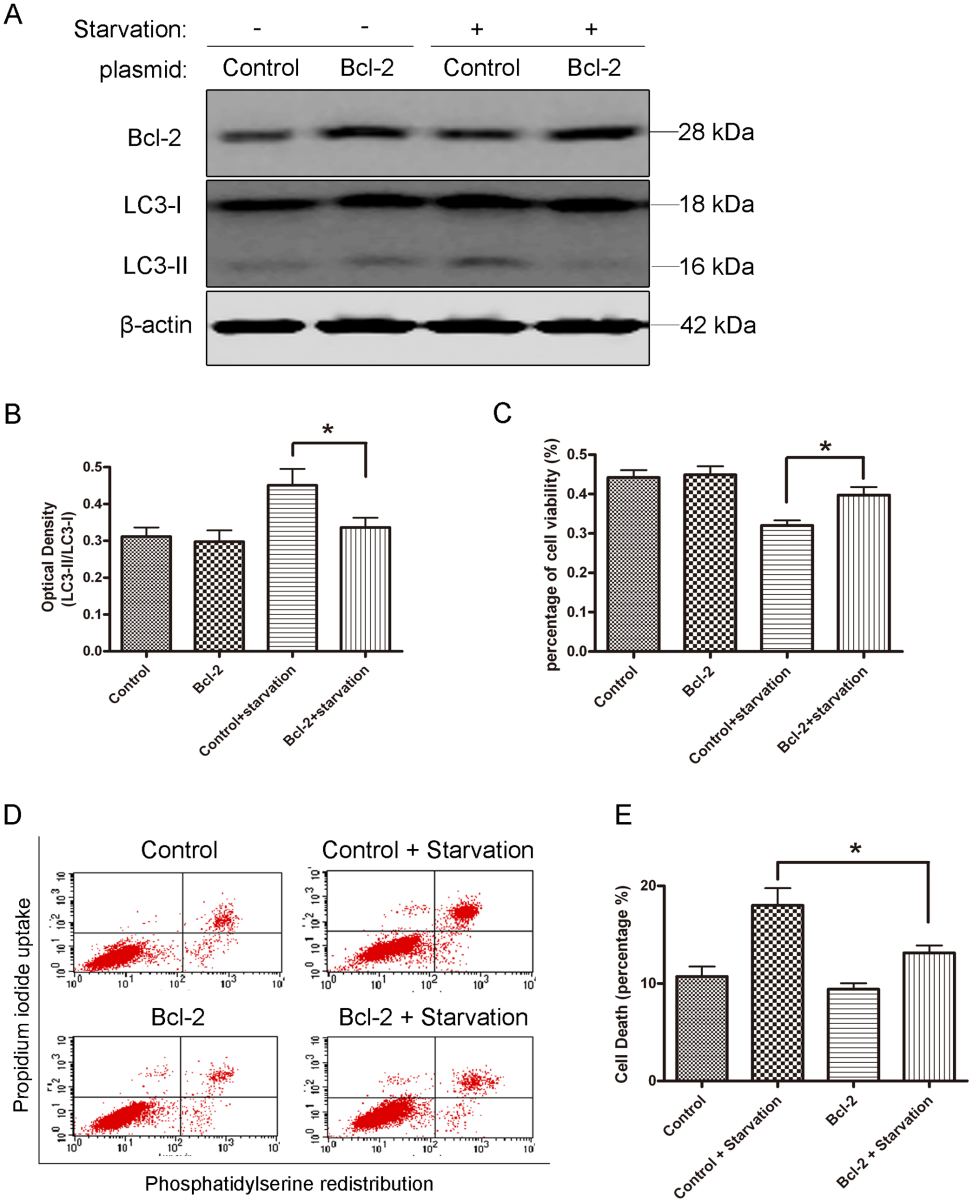
Overexpression of Bcl-2 inhibited autophagy and blocked cell death. A: SH-SY5Y cells were transiently transfected with pCDNA3.1-Bcl-2 or empty control vector, and the cells were subjected to serum starvation for 12 hrs after 48 hrs transfection. B: Quantitative analysis of optical densities of the LC3-II/LC3-I protein bands with Sigma Scan Pro 5. Bars represent Mean ± SD (n = 3). C: Quantitative analysis of cell viability with MTT assay in SH-SY5Y cells 24 hrs after transfection with control vector or Bcl-2 plasmid. Cells were either maintained in normal or starvation medium for 12 hrs prior to cell viability assay. D: Flow cytometry analysis of SH-SY5Y cells transfected with mock control and Bcl-2 plasmid for 48 hrs, then the cells were growing in normal medium or subjected to 24 hrs starvation. E: Quantitative analysis of cell death by flow cytometry Annexin V/PI staining. Statistical analysis was carried out with ANOVA followed by Dunnett t-test (*p<0.05). Data represent mean ± SD for combined data from three independent experiments. For MTT assay, each experiment has six replicate wells.

To further address the role of autophagy in serum-starvation induced cell death, we downregulated essential autophagic gene Beclin1 with siRNA (Fig. 6, A and B). When Beclin1 was knocked down, serum deprivation did not robustly induce LC3-II up-regulation (Fig. 6, C and D), indicating that autophagic activity was severely impaired. Then, SH-SY5Y cells were co-transfected with Bcl-2 siRNA and Beclin1 siRNA. The transfection itself did not affect the cell viability. However, Beclin1 down-regulation rescued large numbers of cells from death induced by Bcl-2 knockdown under nutrient deprivation conditions (12 hrs and 24 hrs) (Fig. 6E). Moreover, apoptotic inhibitor, Z-VAD, did not further enhance cell viability under Beclin1 knockdown conditions. These data strongly suggest that autophagy plays an important role in these types of cell death. To evaluate the effects of Bcl-2 on autophagy, we transiently transfected Bcl-2 into SH-SY5Y cells. Overexpression of Bcl-2 itself did not cause significant autophagic flux change, while inhibited serum starvation induced LC3-II transformation (Fig. 7, A and B), indicating that the formation of autophagosomes was inhibited. Overexpression of Bcl-2 increased the cell viability under serum starvation conditions (Fig. 7C). Annexin V and PI staining also showed that starvation-induced cell death was prevented by overexpression of Bcl-2 (Fig. 7, D and E). Taken together, these results support the concept that Bcl-2 blocks autophagy and cell death under nutrient deprivation conditions.

## Discussion

Autophagy has been reported to either inhibit or promote cell death under different conditions. Anti-apoptotic protein Bcl-2 inhibits autophagy under certain conditions. However, autophagy can also be induced under the condition in the presence of overexpressed Bcl-2/Bcl-xL after ischemia [24] and treated with apoptotic reagents [8]. Although it is consistently demonstrated that autophagy plays a pro-survival role during nutrition starvation, the relationships between Bcl-2 and autophagy and the precise role of Bcl-2 in cell survival under nutrition stress conditions remains to be further characterized. In this study, we showed that serum deprivation-induced autophagy was accompanied by an up-regulation of total and phosphorylated protein levels of Bcl-2 in neuroblastoma SH-SY5Y cells. Bcl-2 knockdown or Bcl-2 inhibitors further enhanced autophagic flux, decreased cell viability and finally led to apoptotic and autophagic cell death under nutrient deprivation condition. The cell death could be blocked by knockdown of essential autophagic gene Beclin1 or overexpression of Bcl-2. Our results suggest that Bcl-2 plays an important role in regulating adequate autophagy activity and promoting cell survival under nutrition stress conditions.

A “rheostat” of Bcl-2-Belin1 complex has been described by Beth Levine’s group [20]. According to this model, autophagy is necessary for homeostasis as an adaptive response to nutrient deprivation and other forms of cellular stress. However, if the levels of autophagy are induced beyond a physiological range, autophagy can contribute to death execution [20]. The relative amounts of Beclin1 and Bcl-2 family proteins govern the threshold for transition from cell survival to cell death [20]. In the present study, we observed that serum starvation induced autophagy activation as evidenced by the increase in conversion of LC3-I to the membrane form LC3-II, the increase in the expression of Beclin1 and the lysosomal cathepsin D, but decreased the autophagy substrate p62. Meanwhile, serum deprivation also increased the Bcl-2 protein levels. As Bcl-2 functions to inhibit autophagy activity through binding to Beclin1, this study tested if Bcl-2 up-regulation is used as a brake for autophagy over-activation under serum deprivation conditions. When Bcl-2 expression was knocked down with siRNA, or its function was inhibited with antagonists, nutrient deprivation-induced autophagy activity was further enhanced. These data argue strongly that Bcl-2 upregulation maintains autophagy activity to the desired levels and limits autophagy from over-activation.

We next speculated that down-regulation of Bcl-2 could lead to autophagy “unchecked” and excessive autophagy might promote cell death through apoptotic and autophagic mechanisms. Our results demonstrated that when Bcl-2 was knocked down or inhibited with antagonists, serum deprivation led to profound cell death. Autophagic inhibitors, Baf A1 and E64D blocked large number of cell death, indicating that autophagy was involved in this death process. Importantly, knockdown essentially autophagy gene Beclin1 blocked cell death under serum starvation conditions, suggesting that autophagy can switch from protective to detrimental. These observations were consistent with a recent study from Robert et al showing that knockdown of Bcl-B, an anti-apoptotic member of Bcl-2 family which binds and inhibits Beclin1 dependent autophagy, triggers autophagic cell death induced by amino acid starvation. The cell death was partially dependent on the autophagy machinery [23]. In contrast to our results that autophagy is involved in cell death, Mohan et al recently reported that autophagy was inhibited and cells died under serum starvation when subjected to anti-cancer drugs, 4-HPR and APG, using neuroblastoma SH-SY5Y cells as a model [38]. The reasons for these different observations may be complicated, because the role of autophagy in physiological conditions may different from its role in non-physiological context, such as during chemotherapy. 4-HPR and APG are not recognized autophagy inhibitors, and these two compounds induce apoptosis of cancer cells by other mechanisms rather than inhibiting autophagy in various cancers. In the present study, we focused on the role of Bcl-2 in serum starvation condition, a classical model of autophagy activation. Our findings demonstrated that under nutrient starvation conditions, down-regulation of Bcl-2 can induce cell death in a manner partially dependent on autophagic genes.

Our observation that no robust cell death occurs under acute serum starvation conditions suggests that the up-regulated protein level of Bcl-2 may be essential to maintain cell viability. Likewise, studies with Bcl-2 knockdown and Bcl-2 inhibitors demonstrated that Bcl-2 is indeed crucial for cell survival under nutrient deprivation conditions. We found that when Bcl-2 was knocked down or inhibited with antagonists, serum deprivation led to the greater loss of cell viability. Flow cytometry analysis showed that starvation caused an insignificant increase in apoptosis. However, apoptosis was greatly increased when Bcl-2 was knocked down, as evidence by inversion of phosphatidylserine, caspase-3 and PARP cleavage. The cell death was partially blocked by pan-caspase inhibitor Z-VAD. These data confirmed that cell death was partially through an apoptotic mechanism.

HA 14-1 is a nonpeptidic ligand of a Bcl-2 surface pocket which mediates anti-apoptotic interactions and triggers apoptosis in Bcl-2-expressing cell lines [39]. ABT-737, a small molecule BH3 domain peptide mimetic, shows synergistic cytotoxicity and causes regression of established tumors [29]. These two compounds act similarly to derepressor/sensitizer BH3 domain-only peptides. Our results showed that separate treatment with the two compounds do not lead to cell death in normal conditions, indicating that inhibition of the function of Bcl-2 is not sufficient to cause cell death. However, in presence of these compounds, nutrition deprivation induced robust cell death. In addition, prolonged serum deprivation (24 hours) produced significant apoptosis and overexpression of Bcl-2 completely rescued apoptosis. These data indicate that Bcl-2 is essential for maintenance of the balance between anti-apoptotic and pro-apoptotic molecules, and the inhibition of Bcl-2 function sensitizes the cells to serum starvation. The concept that Bcl-2 acts as a key player in limiting autophagy in a physiological range has particular significance in the nervous system. Autophagy has been observed in diverse neurodegenerative diseases, including Alzheimer’ disease, Parkinson’s disease and Huntington’s disease abnormal autophagy is considered to contribute to the pathogenesis of these disorders [40]. Interestingly, the levels of autophagy in neurons was reported to be exceptionally efficient and tightly regulated [41]. Therefore, Bcl-2 may be a crucial target for study of the mechanisms underlying abnormal autophagy observed in these neurodegenerative diseases.

In summary, the present study suggests that Bcl-2 up-regulation could limit the over-activation of autophagy and prevent apoptosis under nutrition deprivation stress. The role of Bcl-2 in regulating autophagy and cell survival in other pathological conditions warrant further studies. This may provide more molecular insights into the apparent paradoxical roles of autophagy in cell death and cell survival under different conditions.

## Materials and Methods

### Antibodies, Plasmids and Reagents

Polyclonal anti-Beclin1 (H-300), polyclonal anti-cathepsin D (H-75), monoclonal anti-Bcl-2 (C-2) and monoclonal anti-Bcl-xl (H-5) antibodies were purchased from Santa Cruz Biotechnology (Santa Cruz, CA, USA). Polyclonal anti-LC3 antibody was from Abcam (Cambridge, UK). Phosphor-specific Ser70 rabbit monoclonal anti-Bcl-2 antibody (5H2), polyclonal anti-PARP antibody was from Cell Signaling Technology (Danfoss, MA, USA). Polyclonal anti-caspase 3 antibody was from ENZO life sciences (Farmingdale, NY, USA). Monoclonal anti-p62 and anti-β-actin antibodies were from Sigma (Saint Louis, MO, USA). The Bcl-2 plasmid and empty vector (pCDNA3.1-Bcl-2/pCDNA3.1) was kindly provided by Guanghui Wang (Department of Pharmacology, Soochow University). HA 14-1, rapamycin, ammonium chloride (NH_4_Cl), E64D, bafilomycin A1 (Baf A1) and 3-(4, 5-dimethythiazol-2-yl) 2, 5-diphenylterrazolium bromide (MTT) were from Sigma. ABT-737 was from Selleck Chemicals (Houston, TX, USA). Z-VAD was from Merck chemicals (Darmstadt, Germany).

### Cell Culture

The SH-SY5Y neuroblastoma cell line was from ATCC (Manassas, VA, USA). Cells were grown in a 1∶1 mixture of Ham’s F-12 medium (GIBCO, Gaithesburger, MD, USA) and Eagle’s minimum essential medium (EMEM, ATCC) with nonessential amino acids, supplemented with 10% heat-inactivated fetal bovine serum (GIBCO), penicillin (100 units/ml) and streptomycin (100 µg/ml). Cells were routinely passaged every 5–7 days and cells within 20 passages were used for experiments. For serum starvation, culture medium was removed and cells were rinsed with serum-free medium three times delicately, then added serum-free medium for serum deprivation treatment.

### GFP-LC3 Assay

The SH-SY5Y cells were plated into 24 wells and transiently transfected with GFP-LC3 plasmid for 24 hours, then the cells were fixed in 3.7% paraformaldehyde. The number of GFP-LC3 punctuate dots per cell in GFP-LC3-positive cells were counted applying Nikon D-Eclipse C1 Confocal Laser Scanning Microscope. Thirty cells per slide were counted and three slides were used for each condition.

### RNA Interference and Overexpression

Two sequences for human Bcl-2 double-stranded oligonucleotides (target sequence, #6, 354–372, 5′–GCUGCACCUGACGCCCUUCTT-3′; #7, 1014–1032, 5′-CCCUGUGGAUGACUGAGUATT-3′), for human Beclin1 (target sequence, 5′-AAGAUCCUGGACCGGGUCACC-3′) and a negative control scramble oligonucleotide were synthesized (GenePharma, Shanghai, China). The cells were transfected with siRNA formulated into liposomes (Lipofectamine RNAiMAX, Invitrogen, Burlingame, CA, USA) according to the manufacturer’s instructions. The final concentration is 33 nM for all siRNAs. Cells were harvested for analysis 72 hours after transfection. Transfection efficiency of siRNA in SH-SY5Y cells was estimated with nonspecific FAM-conjugated oligonucleotides and found to be ≥90%. The Bcl-2 plasmid (2 µg), control vector or GFP-LC3 plasmid (0.5 µg) was transfected into the cell using Lipofectamine LTX and PLUS (Invitrogen, Burlingame, CA, USA) according to the manufacturer’s instructions.

### Western Blot Analysis

Western blot analysis was done as described previously [42]. Briefly, protein concentration was determined using a BCA kit (Pierce Biotechnology, Waltham, MA, USA). The proteins were separated by SDS-PAGE and then transferred to nitrocellulose membrane (Bio-Rad Laboratories, Hercules, CA, USA). The antibody dilutions were as follows: anti-Beclin1, 1∶1000; anti-LC3, 1∶2000; anti-cathepsin D, 1∶400; anti-p62, 1∶2500; anti-Bcl-2, 1∶500; anti-Bcl-xl, 1∶500; anti-caspase, 1∶1000; anti-actin, 1∶10000. Immunoreactivity was either detected with an ECL kit (Amersham Biosciences, Arlington Heights, Illinois, USA) and expose to film (Kodak, Rochester, NY, USA), or incubated the membrane with fluorescent secondary antibody (IRDye, LI-COR, Lincoln, NE, USA) and scanned with Odyssey® Western Blot Analysis system (LI-COR, Lincoln, NE, USA). The signal intensity of primary antibody binding was quantitatively analyzed with Sigma Scan Pro 5 and was normalized to a loading control β-actin.

### Cell Viability and Apoptosis Assay

For siRNA and overexpression, SH-SY5Y cells were seeded into 96-well plates (Thermo-Fisher Scientific, Waltham, MA, USA) at a density of 1×10^5^/ml, transfection was performed at ∼80% confluence, 24 hours (overexpression) or 72 hours (siRNA) after transfection, the cells were starved for another 12 or 24 hours. For HA 14-1 and ABT-737 treatments, SH-SY5Y cells were seeded into 96-well plates at the density of 1×10^5^/ml, 12 hours after sub-culture, cells were pre-treated with 5 µM HA 14-1 or 100 nM ABT-737 for 30 minutes. Then cells were either grown in normal media and starvation media in the presence of DMSO, HA 14-1 or ABT-737 for another 12 or 24 hours. In brief, MTT was added to the medium at a final concentration of 5 mg/ml for 1 hour at 37°C and 5% CO_2_. Then medium was removed and 100 µl of DMSO were added and shaken for 15 minutes. One well contained 100 µl of DMSO alone as a blank. The absorbance of the wells was read at 570 nm using a spectrophotometer. For apoptosis assay, each sample was harvested and stained with Annexin V and Propidium iodide (Beyotime Institute of Biotechnology, Nantong, China), then the cells were analyzed with flow cytometric analysis (*Beckman* Coulter Cytomics *FC500*, Brea, CA, USA).

### Statistical Analysis

Statistical analysis was carried out either by one-way ANOVA followed by Dunnett’s t-test or by two-tailed unpaired t-test. Differences were considered significant when P<0.05.
